# Ebola, the killer virus

**DOI:** 10.1186/s40249-015-0048-y

**Published:** 2015-04-08

**Authors:** Haider Ghazanfar, Fizza Orooj, Muhammad Ahmed Abdullah, Ali Ghazanfar

**Affiliations:** Shifa College of Medicine, Pitras Bukhari Road H-8/4, Islamabad, Pakistan; Federal Medical and Dental College, Prime Minister’s National Health Complex, Chak Shahzad, Islamabad, Pakistan

**Keywords:** Ebola hemorrhagic fever, Epidemiology, Ebola virus/physiology, Hemorrhagic fever, Ebola/transmission, Ebola/prevention and control

## Abstract

Ebola virus disease (EVD) has mostly affected economically deprived countries as limited resources adversely affect a country’s infrastructure and administration. Probing into the factors that led to the widespread outbreak, setting forth plans to counter EVD cases in developing countries, and devising definitive measures to limit the spread of the disease are essential steps that must be immediately taken. In this review we summarize the pathogenesis of EVD and the factors that led to its spread. We also highlight interventions employed by certain countries that have successfully limited the epidemic, and add a few preventive measures after studying the current data. According to the available data, barriers to prevent and control the disease in affected countries include irresolute and disorganized health systems, substandard sanitary conditions, poor personal hygiene practices, and false beliefs and stigma related to EVD. The public health sector along with the respective chief authorities in developing countries must devise strategies, keeping the available resources in mind, to deal with the outbreak before it occurs. As a first step, communities should be educated on EVD’s symptoms, history, mode of transmission, and methods of protection, including the importance of personal hygiene practices, via seminars, newspapers, and other social media. A popular opinion leader (POL) giving this information would further help to remove the misconception about the nature of the disease and indirectly improve the quality of life of affected patients and their families.

**Electronic supplementary material:**

The online version of this article (doi:10.1186/s40249-015-0048-y) contains supplementary material, which is available to authorized users.

## Multilingual abstracts

Please see Additional file 1 for translation of the abstract into the six official working languages of the United Nations.

## Introduction

**Figure 1:**
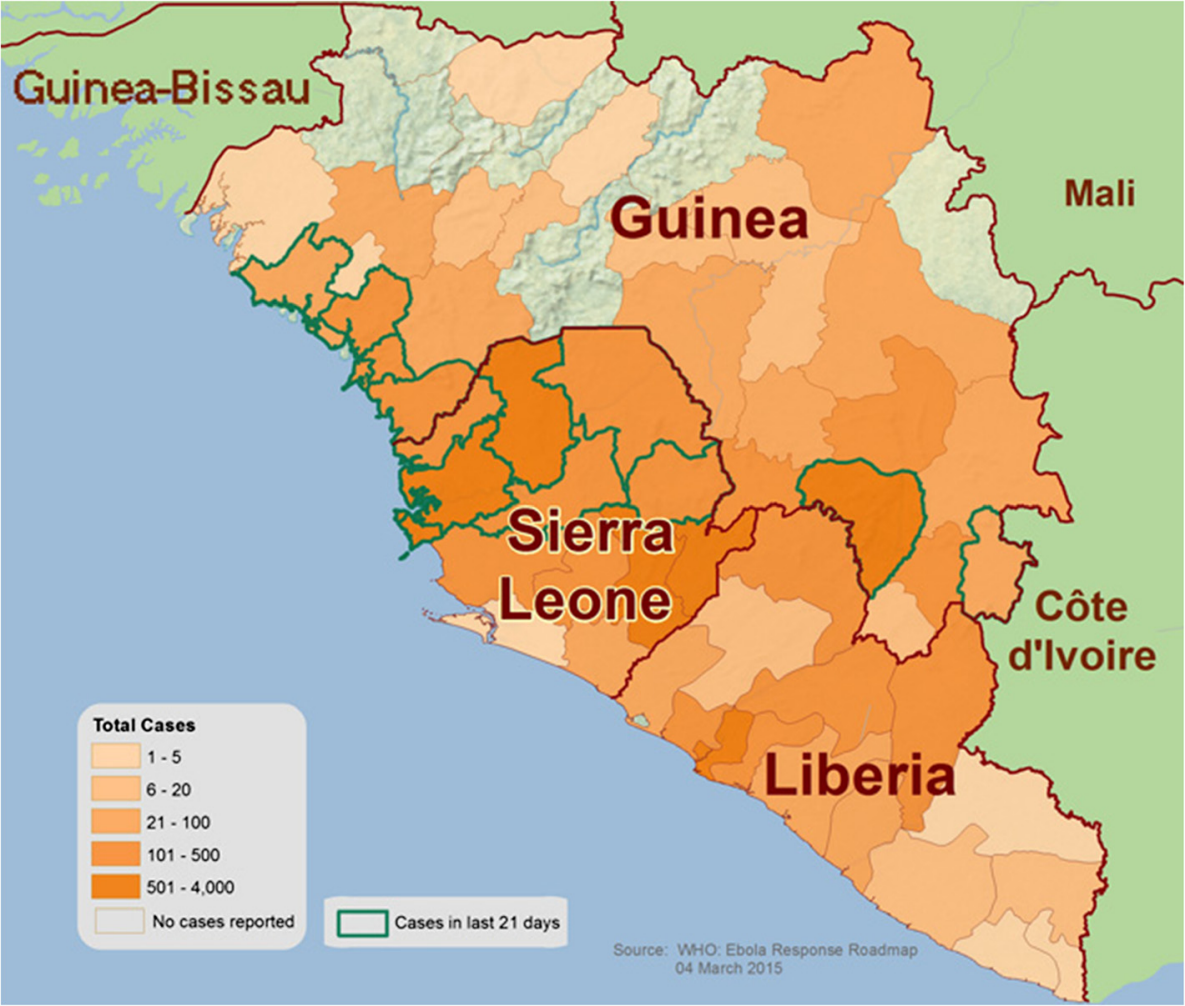
**The 2014 Ebola outbreak in West Africa.**

The first Ebola virus disease (EVD) outbreak occurred simultaneously in Nzara, Sudan (involving 281 patients out of which 151 died [54%]) [1] and Yambuku, Zaire (now the Democratic Republic of Congo) (involving 318 patients out of which 280 died [88%]) [2] in 1976. The disease got its name from the Ebola River, which passes near the Yambuku village where the outbreak first occurred [3]. The first case of the current EVD outbreak in West Africa was reported in Guinea in March 2014 [4], and from there it spread across land borders to Liberia and Sierra Leone, and to Senegal (by land travel) and Nigeria (by air travel) [5,6]. The World Health Organization (WHO) declared it a “Public Health Emergency of International Concern” on August 7, 2014 [6]. Ebola virus disease has an average case fatality rate of 50% [6]. As of February 4, 2015, a total of 22,500 confirmed, probable, and suspected cases of EVD, and almost 9,000 deaths have been reported [7]. A total of 132 new confirmed cases were reported in the week ending to March 1 [8]. The case fatality rate of the current outbreak in Guinea, Liberia and Sierra Leoneis 76%; it is slightly less (61%) in hospitalized patients [9]. Research has shown that EVD has mostly affected economically deprived countries as limited resources adversely affect a country’s infrastructure and administration [10]. Probing into the factors that led to the widespread outbreak, setting forth plans to counter EVD cases in developing countries, and devising definitive measures to limit the spread of the disease are essential steps that must be immediately taken (Figure 1).

## Review

We conducted a narrative clinical review on the epidemiology, transmission, clinical manifestation, diagnosis, and prevention of Ebola virus. We searched for online articles using MEDLINE® and Google Scholar. The keywords/phrases used were: “Ebola virus disease”, “Epidemiology of Ebola virus disease”, “Mortality and morbidity associated with Ebola virus disease”, “Pathogenesis of Ebola virus disease”, “Clinical manifestation of Ebola virus disease”, and “Prevention of Ebola virus disease”. Articles dated from 1976 to 2015 were included in the study. Articles were reviewed from November 2014 to March 2015. Search results yielded a total of 174 articles out of which 52 articles were included in this study.

### What is Ebola virus?

Ebola virus belongs to the family *Filoviridae*, which compromises three genera: *Cuevavirus*, *Marburgvirus*, and *Ebolavirus***.** This family belongs to the order *Mononegavirales***.** Five species of Ebola virus have been identified: Zaire, Sudan, Bundibugyo, Reston, and Taï Forest. *Bundibugyo ebolavirus*, *Zaire ebolavirus*, and *Sudan ebolavirus* have been associated with large outbreaks in Africa. The current West African outbreak was caused by *Zaire ebolavirus*, which is also the most virulent among the five species. Since 1976 it has caused multiple outbreaks in Central Africa, with a mortality rate of 55%–88% [11-14]. The Ebola virus genome contains a single strand of non-segmented, negative-sense viral genomic RNA [15].

### How is Ebola virus transmitted?

Pteropodidae families of fruit bats are thought to be the natural reservoirs of Ebola virus [16]. Humans can be infected by Ebola virus by direct contact with blood and body fluids of infected animals such as apes, gorillas, fruit bats, and monkeys [6,17,18]. There is no evidence that pet cat and dogs, mosquitoes, or other insects can transmit Ebola virus [17]. Human-to-human transmission occurs via direct contact with the blood, organs, secretion, and other bodily fluids (such as urine, feces, semen, breast milk, mucus, vomit) of an infected person, and via surface and materials contaminated with these fluids [6,19-21]. Infected syringes and needles are other ways by which the virus can be transmitted from an infected human to uninfected humans [17]. The Ebola virus does not spread through air or water [17]. Breaches in infection control precautions have resulted in frequent infections of health workers treating this outbreak. Direct contact with the body of a deceased person during burial ceremonies is another way by which Ebola virus can be transmitted [22].

The Center for Disease Control and Prevention (CDC-US) and the WHO have recommended that infected individuals should be quarantined for 21 days. The incubation period of Ebola virus is 2–21 days after infection. Latest studies have shown that Ebola virus transmission occurs when there is a high viral load of bodily fluids [23]. The person remains infectious as long as the virus is present in their blood and bodily fluids. Patients who have completely recovered from the Ebola virus cannot spread it. Ebola virus has been detected in semen of recovered patients, but it’s not known yet if it can spread through sex. The WHO advises to abstain from sex or use condoms for a period of three months after the patient is cured [6]. There is no evidence yet on when women recovering from the Ebola virus can resume breastfeeding in [24].

### What are the clinical symptoms of EVD?

The initial symptoms of EVD include fever, headache, fatigue, sore throat, and muscle pain, which are followed by anorexia, nausea, diarrhea, vomiting, rash, abdominal pain, cough, shortness of breath, postural hypotension, edema, headache, confusion, and coma [25]. In some cases, a maculopapular rash develops after 5–7 days of symptoms [26]. In severe cases, the patient also develops hemorrhagic complications (such as mucosal hemorrhages, nose bleeding, vomiting/coughing up blood, blood in stool, petechiae, ecchymoses, uncontrollable bleeding from venipuncture sites), severe metabolic disturbances, convulsion, shock, and multiple organ failure. These complications are the most common causes of death in patients [25]. Symptoms can appear anywhere between 2–21 days [27]. Gastrointestinal symptoms are the most common in the current outbreak [28].

### How is EVD diagnosed?

Ebola virus usually reaches detectable levels in blood after three days of symptoms [29]. A negative test before this does not rule out EVD. IgM enzyme-linked immunosorbent assay (ELISA), antigen-capture ELISA, polymerase chain reaction (PCR), and virus isolation are the diagnostic tests available to diagnose a patient who presents at a health facility within a few days of showing symptoms [29]. IgM and IgG antibodies are used for diagnosis later in the disease course or after recovery. Laboratory findings in EVD include Leukopenia, thrombocytopenia, and elevated liver enzyme. Early and well-regulated inflammatory response with elevated IL-6 concentration and IL-1beta presence in a symptomatic patient is indicative of a good outcome, while a defective innate immune reaction with excessive macrophage/monocyte activation with release of interleukin-10, absent antibody response and elevated concentration of interleukin-1RA, and neopterin after a few days of onset of disease is associated with a fatal outcome [30]. According to one study, lymphoid depletion and lymphopenia associated with *Zaire ebolavirus* is most likely due to lymphocyte apoptosis via Fas/FasL interaction [30]. The excessive macrophage/monocyte activation leads to a “cytokine storm” triggering disseminated intravascular coagulation, hypotension, and vascular dysfunction, resulting in multiple organ failure, vascular collapse, and shock [31]. According to a recent study, elevated thrombomodulin and ferritin levels have been associated with death and hemorrhage in Ebola virus infected patients [32].

### How to prevent the spread of EVD?

According to an estimate, 2–8 patients are exported from the three major Ebola-inflicted countries in Africa monthly, with 64% of the destinations being economically deprived countries [33]. This poses (still unidentified) problems for communities, which might get affected by Muslims travelling from these countries for Hajj, the annual Muslim pilgrimage to Mecca. Screening at all the export portals of the three afflicted countries would be the most effective way of containing the outbreak. However, implementing such screening protocols is beyond the abilities of any individual country without international assistance. One thing that can be implemented, however, is the banning of all transportation from the affected countries, not only via air travel but also via ships. A study in China calculated the number of imported cases of EVD using the basic reproductive number (R0) and found that early interventions led to a substantial decrease in the prevalence and duration of the epidemic [34]. Hence, urgent measures need to be taken so that all predominantly Muslim countries are prepared to combat this epidemic.

According to the available data, barriers to prevent and control the disease in affected countries include irresolute and disorganized health systems, substandard sanitary conditions, poor personal hygiene practices, and false beliefs and stigma related to EVD [35]. There are further hindrances due to the unavailability of electricity, water, adequate communication services between health officials, and poor facilities for transportation of patients and specimens [36]. The public health sector along with the respective chief authorities in developing countries must devise strategies, keeping the available resources in mind, to deal with the outbreak before it occurs.

As a first step, communities should be educated on EVD’s symptoms, history, mode of transmission, and methods of protection, including the importance of personal hygiene practices, via seminars, newspapers, and other social media. A popular opinion leader (POL) giving this information would further help to remove the misconception about the nature of the disease and indirectly improve the quality of life of affected patients and their families [37,38]. In addition, health systems should formulate proper plans for emergency care, ensuring adequate quarantine facilities, proper surveillance, case management, and contact tracing. Training should be given to healthcare providers in areas such as prompt diagnosis and isolation of a suspected patient, the importance of wearing personal protective equipment, and safe burial techniques [36]. There should be adequate distribution of gloves, gowns, masks, soaps, and disinfectants to healthcare facilities, and safety precautions should be devised especially for laboratory personnel including pre-transfusion testing [39]. The CDC guidelines for monitoring patients (including symptomatic and asymptomatic), and precautions for healthcare professionals (including wearing personal protective equipment, practicing personal hygiene, use of disposable medical instruments, minimizing pricking and aerosol producing procedures, monitoring exposed staff, and adequate environment control) should be practiced [40]. Special ambulances should be reserved to enable the safe transport of EVD patients.

At state level, funds for proper sanitation, especially in rural areas, should be reserved. Measures should be taken to ensure the availability of water and electricity in all areas. Internet and telephone services should be made available round the clock in all hospitals and healthcare facilities for effective communication, and for reporting and enrolling EVD cases. Home protective kits can further enhance control measures, thus their distribution at household-level should be considered [41].

Incident management systems (IMSs), such as the one adopted by the CDC for the control of the current epidemic, have proven efficacious in preventing the spread and adequately controlling the disease [42,43]. A report about the employment of an IMS divulged that Nigeria has successfully limited the outbreak and no further cases have been reported since August 31, 2014 [43]. The employment of such a system has resulted in a decrease of EVD patients in Liberia [44]. Thus, an IMS may be adopted and modified keeping in view the available resources and infrastructure of the respective country. The system involves authorizing representative health personnel to fulfill specific tasks including international correspondence, setting forth important measures to respond to the event (including surveillance), supporting affected families, devising plans to curtail chaos among the public, and monitoring healthcare providers [41]. The CDC also recommends limiting the movement of patients (restriction of public transport especially aircrafts, ships, trains, and buses), which states should implement if EVD strikes their nation [45]. Rapid identification and treatment of EVD-infected patients can significantly reduce the possibility of secondary transmission [46].

Many drugs are being probed as preventive medications for EVD, such as amiodarone, chloroquine, and clomiphene [37]. An effective vaccine is also being devised; recombinant vesicular stomatitis virus vaccine has been the most promising, yet its efficacy has so far not been tested in humans [47-50]. Another study found that virus-like particles (VLPs) can provide post-exposure protection by amplifying Type 1 interferon signaling in macrophages and dendritic cells, which are thought to be the initial Ebola virus infection sites [51]. One study showed that even if there was a vaccine, when the nature of the disease is considered, vaccination wouldn’t be efficacious and cost-effective in controlling the current outbreak, and would divert the attention of health authorities from proven effective control measures [52]. However, vaccination can reduce case fatality and prevent further outbreaks. Another problem is that Ebola virus mostly affects low-income countries, which might not have enough resources to develop a vaccine. However, as EVD is considered a weapon of bioterrorism, this may drive the developed world to work out a definitive and safe vaccine soon enough. Until then, countries should concentrate on early detection, isolation, prompt treatment, contact tracing, and proper burials.

## Conclusion

The public health sector along with the respective chief authorities in developing countries must devise strategies, keeping the available resources in mind, to deal with the outbreak before it occurs.

## Additional file

Additional file 1:
**Multilingual abstracts in the six official working languages of the United Nations.**
