# Supplementary material for: Ebola, the killer virus
Source: Infect Dis Poverty. 2015 Apr 8;4:15. doi: 10.1186/s40249-015-0048-y (PMC4393611; doi:10.1186/s40249-015-0048-y)
Supplement: Additional file 1: — Multilingual abstracts in the six official working languages of the United Nations. [file 40249_2015_48_MOESM1_ESM.pdf]

Translation of the abstract into the six official working languages of the United Nations

إيبولا- الفيروس القاتل

حيدر غضنفر، الدكتورة فائزة أروج، أحمد عبد الله، علي غضنفر

ملخص
لقد أضر مرض فيروس إيبولا((EVD) بالأساس دولا محرومة اقتصاديا باعتبار محدودية مواردها التي أثرت سلبا على بنيتها التحتية وإدارتها. يعتبر التحقيق في العوامل التي أدت إلى انتشار المرض على نطاق واسع، ووضع خطط لمقاومة حالات الإصابة بمرض فيروس إيبولا في الدول النامية، إضافة إلى اتخاذ تدابير حاسمة للحد من انتشار هذا المرض، من المراحل الأساسية التي يجب التي يجب اتخاذها حالا.
سنلخص في هذه المراجعة كيفية نشوء مرض فيروس إيبولا (EVD) والعومل التي أدت إلى انتشاره. كما سنسلط الضوء على التدخلات التي قامت بها بعض الدول والتي ساهمت بنجاح في الحد من انتشار المرض. كما سنضيف بعض التدابير الوقائية بعد دراسة المعطيات الحالية. حسب المعطيات المتوفرة، تضم الحواجز التي تساهم في الوقاية من المرض ومراقبته في الدول المتضررة في وجود أنظمة صحية غير صارمة وغير منظمة، وظروف صحية متدنية، وعادات متدنية في النظافة الشخصية. يجب على قطاع الصحة في الدول النامية والهيئات العليا الخاصة بها أن تضع استراتيجية مع الأخذ بعين الاعتبار الموارد المتاحة كي تتمكن من التعامل مع الوباء قبل انتشاره. يجب في مرحلة أولى تثقيف المجتمعات بأعراض مرض فيروس إيبولا، وبطرق انتقاله،
وسبل الوقاية منه، بما في ذلك أهمية عادات النظافة الشخصية، وذلك من خلال الندوات والصحف ووسائل الإعلام الاجتماعية الأخرى. إن تقديم هذه المعلومات من قبل قائد شعبي (POL) من شأنه أن يساهم في إزالة سوء الفهم المرتبط بطبيعة هذا المرض كما سيحسن بطريقة غير مباشرة من نوعية حياة المرضى المصابين وأسرهم.

Translated from English version into Arabic by malika2012, through

TRANSLATORS
WITHOUT BORDERS

致命病毒——埃博拉

Haider Ghazanfar, Fizza Orooj, Ahmed Abdullah, Ali Ghazanfar

摘要
因为资源匮乏阻碍国家基础设施建设和行政管理，埃博拉病毒病（EVD）对贫困国家的影响很大。因此，必须立即探索埃博拉病毒大肆流行的原因，制定计划以遏制该病在发展中国家的传播，并实施明确的方案，阻止疾病扩散。本文总结了埃博拉的发病机制及其传播诱因，重点强调了特定国家成功遏制疫情的有效干预措施，同时通过现有资料补充了一些预防措施。从所得资料来看，疫区国家防控疾病的障碍包括组织不力的卫生系统，卫生条件较差，不良的个人卫生习惯和对埃博拉及其相关病毒的错误认识。发展中国家的公共卫生部门以及其他主要相关部门必须多方考虑，运筹帷幄，将疫病爆发扼杀在摇篮里。首先，必须通过研讨会、报纸和其他社会媒体向社区居民传授埃博拉的相关知识，如：它的流行特征、发展历史、传染方式、防护措施，包括个人卫生习惯的重要性。主流大众舆论倡导这样的信息将会进一步帮助人们消除对这种疾病本质的误解，并间接提高患者及其家属的生活质量。

Translated from English version into Chinese by Chen Jin, edited by Yin Jian-hai, through

CDC CHINESE CENTER FOR DISEASE CONTROL AND PREVENTION
NATIONAL INSTITUTE OF PARASITIC DISEASES

Ebola, le virus qui tuer

Haider Ghafanzar, Fizza Orooj, Ahmed Abdullah, Ali Ghazanfar

Résumé
Les pays touchés par la maladie à virus Ebola (MVE) sont, en général, des pays économiquement défavorisés puisque des ressources limitées portent préjudice aux infrastructures et à l'administration de ces pays. Des mesures doivent être prises immédiatement afin de limiter la propagation du virus : il faut se pencher sur les facteurs ayant mené à l'épidémie généralisée de MVE, établir des plans visant à contrer les cas de MVE dans les pays en développement, ainsi qu'élaborer des mesures définitives pour enrayer la propagation de la maladie. Dans cette analyse, nous résumerons la pathogenèse de la MVE, nous mettrons en lumière les interventions mises en œuvre dans certains pays ayant réussi à contenir l'épidémie, et nous proposerons quelques mesures de prévention découlant de l'étude des données actuelles. Selon les données disponibles, les facteurs suivants empêchent la prévention et le contrôle de la maladie dans les pays touchés : un système de santé désorganisé et sans réelle direction, des conditions sanitaires déplorables, une hygiène personnelle déficiente ainsi que des croyances erronées et des préjugés à propos de la MVE. Le secteur de la santé publique, en collaboration avec les autorités responsables des différents pays en développement, doivent mettre au point des stratégies, selon les ressources disponibles, afin de faire face à l'épidémie avant qu'elle ne survienne. La première étape demeure la sensibilisation des collectivités aux symptômes liés à la MVE, à l'histoire de la maladie, à son mode de transmission ainsi qu'aux moyens de s'en protéger (en insistant sur l'importance d'une bonne hygiène personnelle) dans les journaux, les séminaires ou dans d'autres médias

sociaux. La transmission de ces informations par un leader d'opinion aiderait d'autant plus à dissiper les fausses idées véhiculées à propos de la MVE et améliorerait indirectement la qualité de vie des personnes atteintes et de leur famille.

Translated from English version into French by Chantal Quintric Leveille, through

Эбола, вирус-убийца

Хайдер Газанфар, Фицца Оруж, Ахмед Абдулла, Али Газанфар

Реферат
Болезнь, вызванная вирусом Эбола (БВВЭ), в основном поразила экономически отсталые страны, поскольку ограниченность ресурсов отрицательно сказывается на развитии инфраструктур страны и управлении на всех уровнях. Необходимо незамедлительно предпринять следующие важнейшие меры: изучить факторы, которые привели к массовым вспышкам болезни, разработать программы борьбы с БВВЭ в развивающихся странах и определить план мероприятий по ограничению распространения болезни. В данном обзоре рассматриваются механизмы развития БВВЭ и факторы, которые привели к ее распространению. Освещаются осуществленные некоторыми странами мероприятия, позволившие успешно ограничить распространение эпидемии, и на основании изучения имеющихся данных предлагается ряд профилактических мер. В соответствии с имеющимися данными основными препятствиями в профилактике и борьбе с болезнью в пораженных ею странах являются хаотичные и неорганизованные системы здравоохранения, неудовлетворительные санитарные условия и личная гигиена, ложные убеждения и предрассудки, связанные с БВВЭ. Сектора здравоохранения и соответствующие органы власти развивающихся стран должны разработать стратегию борьбы со вспышкой болезни до ее начала и с учетом всех имеющихся ресурсов. Прежде всего посредством организации семинаров, через печать и социальные сети необходимо ознакомить население с симптомами БВВЭ, ее историей, путями передачи и способами защиты, включая важность соблюдения правил личной гигиены. Если такая информация будет распространяться авторитетным лидером общественного мнения, это поможет уничтожить ложные представления о характере болезни и улучшить качество жизни заразившихся людей и их семей.

Translated from English version into Russian by Alena Hrybouskaya, through

Ébola, el virus asesino

Haider Ghazanfar, Fizza Orooj, Ahmed Abdullah, Ali Ghazanfar

Resumen
El virus del Ébola (VE) ha afecto en gran medida a los países económicamente débiles, ya que los recursos limitados afectan de forma adversa a la infraestructura y administración de un país. La investigación de los factores que llevaron a la epidemia, el establecimiento de planes para contrarrestar los casos de VE en los países en desarrollo, y la elaboración de medidas definitivas para limitar la propagación de la enfermedad son todos pasos fundamentales que se deben adoptar de inmediato. En este documento resumimos la patogenia del VE y los factores que llevaron a su propagación. También destacamos las intervenciones realizadas por algunos países que han limitado con éxito la epidemia, y agregamos unas cuantas medidas de prevención después de haber estudiado los datos actuales. De acuerdo a los datos disponibles, las barreras para prevenir y controlar la enfermedad en los países afectados incluyen los sistemas de salud indecisos y desorganizados, condiciones sanitarias deficientes, malas prácticas de higiene personal y falsas creencias y estigma relacionado con el VE. El sector de la sanidad pública junto con las autoridades correspondientes en los países en desarrollo deben desarrollar estrategias, teniendo en cuenta los recursos disponibles, para tratar la epidemia antes de que esta suceda. Como primer paso, las comunidades deben ser educadas en los síntomas, historia, modo de transmisión y métodos de protección contra el VE, incluyendo la importancia de las prácticas de higiene personal, a través de seminarios, periódicos y otros medios sociales. Un líder de opinión popular (LOP) que presente esta información ayudará aún más a mejorar el concepto erróneo de la naturaleza de la enfermedad y mejorará indirectamente la calidad de vida de los pacientes infectados y sus familias.

Translated from English version into Spanish by Lizette Britz, through
